# The microbiome in health and disease: a new role of microbes in molecular medicine

**DOI:** 10.1007/s00109-016-1499-8

**Published:** 2016-12-17

**Authors:** Ingo B. Autenrieth

**Affiliations:** 1 Institute of Medical and Hygiene, University Hospital Tübingen, Elfriede Aulhorn Str. 6, 72076 Tübingen, Germany; 2 Interfaculty Institute of Microbiology and Infection Medicine, Tübingen, Germany; 3 German Center for Infection Research (DZIF), partner site Tübingen, Tübingen, Germany

For more than a hundred years, medicine was profoundly influenced by the groundbreaking work of Robert Koch [1] and others demonstrating microbes as causative agents of disease (era Koch 1.0, Table 1). Subsequently, novel treatment and prevention strategies including antimicrobial agents, vaccines, and immunotherapies and the implementation of antiseptic and hygiene measures were developed and proved highly effective in combating infectious diseases. As the field of molecular genetics proceeded, molecular equivalents to the Koch-Henle postulates claimed by Falkow [2] have defined the genetic and molecular principles of microbial pathogenesis (era Koch 2.0, Table 1). At the same time, diagnostic microbiology became highly sophisticated in developing methods to isolate pathogens from microbiota-contaminated samples. The microbiota itself, however, remained largely unexplored and elusive. Despite the well-established notion that humans are colonized by a microbiota with important functional roles such as vitamin synthesis, digestion, and colonization resistance against enteric pathogens [3], the global role of the microbiota in health and disease was until recently largely neglected due to the lack of appropriate analytical methods.

**Table 1:** Progress since the Koch era

**Koch 1.0: Microbes are causative agents of disease (1884)** •The microorganism must be found in abundance in all organisms suffering from the disease but should not be found in healthy organisms. •The microorganism must be isolated from a diseased organism and grown in pure culture. •The cultured microorganism should cause disease when introduced into a healthy organism. •The microorganism must be reisolated from the inoculated, diseased experimental host and identified as being identical to the original specific causative agent.
**Koch 2.0: Genetic and molecular principles of microbial pathogenesis (Falkow 1988)** •The phenotype or property under investigation should be associated with pathogenic members of a genus or pathogenic strains of a species. •Specific inactivation of the gene(s) associated with the suspected virulence trait should lead to a measurable loss in pathogenicity or virulence. •Reversion or allelic replacement of the mutated gene should lead to restoration of pathogenicity.
**Koch 3.0: Metagenomic equivalents of Koch postulates applied to noninfectious pathobiont-associated diseases** •A single strain, species, genus or phylum, or various combinations thereof, or genomically encoded functions, are significantly correlated with the disease phenotype of a host. •Microbiota transfer into an appropriate gnotobiotic defined animal model causes at least some aspects of the disease phenotype or a corresponding change of the metabolome or immune response. •The qualitative or quantitative metagenome alteration should be reported in the newly diseased host. •Depletion of the identified taxa or functions, by intervention via e.g., antibiotics, expansion of beneficial microbes by probiotics, prebiotics, microbiota transfer, or diet reduces progression or ameliorates the disease. •Transmissibility is not essentially required as diseases are often the results of a shift of the abundance of endogenous members/functional capacities of the microbiota.

Although molecular methods like polymerase chain reaction, Sanger sequencing, and fluorescence in situ hybridization techniques brought a deeper insight into the composition of the intestinal microbiota, it turned out that many members of the microbiota cannot be cultivated with current laboratory methods [4]. Experimental approaches tackling the role of microbes in disease, however, largely depend on the ability to cultivate (according to Koch’s postulates) and to genetically manipulate microbes (according to Falkow’s molecular equivalents) in order to test them in experimental systems. The rising field of mucosal immunology together with the development of germ-free and gnotobiotic mouse models, however, attracted attention and provided the tools necessary to address the role of commensal microbes as putative disease-associated opportunistic pathogens or “pathobionts”. Pathobionts are resident microbes innocuous to the host under normal conditions but with pathogenic potential in susceptible hosts (e.g., genetic risk factors). However, the absence of appropriate experimental methods to determine and to analyze the noncultivable microbes from the microbiota had hampered further elucidation of their role in etiology and pathogenesis of diseases.

A breakthrough was achieved by the implementation of the next generation sequencing and new bioinformatic algorithms for metagenomic data analysis [5–7] and provided the foundation for more sophisticated exploration of the microbiota. Consequently, an enormous number of publications reported on the composition and changes of the microbiota, designated as microbiomes of specific body sites, and raised questions and new hypotheses of their roles in health and disease. In fact, nowadays microbes are not only considered as causative pathogens of infectious diseases but also as causative or contributing agents of noninfectious, particularly chronic diseases like allergies, chronic inflammatory bowel diseases, colon cancer, diabetes, and neurodegenerative diseases [8]. Recent achievements to a better understanding of the metabolic function of the microbiome and its role in shaping the immune system shed new light on the mechanisms governing the balance between tolerance, immune effector mechanisms, and inflammatory reactions [9, 10]. Nonetheless, technical limitations of analytic and pre-analytic procedures, experimental setups, and study designs of many studies had hampered the ability to distinguish if composition and changes of the microbiomes are a cause or consequence of disease. In turn, metagenomic shotgun sequencing, humanized gnotobiotic animal models, population-based or long-term patient cohort epidemiologic studies provide suitable tools to test hypotheses and to design proof of concept studies to explore the causative role of single members or communities of the microbiota in aetiopathogenesis of noninfectious diseases.

Despite these achievements, comprehensive analytic pipelines including targeted and nontargeted metabolomic analysis of body fluid compartments and the linkage to metagenomic sequencing data and disease phenotypes are required for diagnostics directing therapeutic interventions via manipulations of the microbiome. To this end, new postulates (era Koch 3.0) based on metagenomics and new analytic tools are necessary (Table 1) to explore and to prove the impact of single microbes or of the concerted action of microbial communities and their metabolites on chronic, noninfectious diseases in the context of an individual host genome with an individual lifestyle and environment.

In the present issue of the *Journal of Molecular Medicine*, several important aspects of current research around the microbiome and its role in human health and disease are discussed: Johnson and colleagues elaborate on the specific components of the microbiota, the *Bacteroidetes*, by discussing the genetic variability of this phylum, the sometimes opposing effects on metabolic diseases such as obesity or type II diabetes and the problem of health-interpretable microbiome data [11]. Yiu and colleagues extend this by reporting recent findings and ideas how host metabolism, body weight, and diseases like obesity may be affected by interactions with the immune system, metabolism, and the microbiota [12]. Referring to chronic inflammatory bowel disease, Frick and Wehkamp discuss current strategies to shape mucosal immunity and the microbiome by innovative therapeutic interventions [13], while Lee et al. discuss new molecular aspects of the gut-brain axis exemplified by how the microbiota affects the cytoplasmic ligand-induced aryl hydrocarbon receptor, AhR, and how this in turn may affect host physiology and diseases including neurodevelopmental and neurodegenerative diseases [14]. Finally, Willmann and Peter address how the rapidly increasing antimicrobial resistance of bacterial pathogens can be tackled by exploring the resistome of the microbiome with the next generation sequencing methods and bioinformatic algorithms and how clinical diagnostics and infection control may profit from molecular microbiota analysis [15].

Future textbooks in human physiology will likely include comprehensive chapters on microbiomes in human health, and future textbooks in clinical medicine will possibly deal with microbes not only in the chapter of classical infectious diseases (caused by typical pathogens) but also in noninfectious diseases (caused or driven by pathobionts). Additionally, they may include how modification of the microbiota by e.g., novel antimicrobial agents, smart and individualized probiotics, prebiotics, small molecules, and lifestyle intervention, or by microbiota transfer may offer new opportunities for the preservation of health and cure of disease. In any case, these are exciting times for microbiology within molecular medicine that herald a new dimension in the concepts of precision medicine.
